# LOTUS, an endogenous Nogo receptor antagonist, is involved in synapse and memory formation

**DOI:** 10.1038/s41598-021-84106-y

**Published:** 2021-03-03

**Authors:** Ryohei Nishida, Yuki Kawaguchi, Junpei Matsubayashi, Rie Ishikawa, Satoshi Kida, Kohtaro Takei

**Affiliations:** 1 Molecular Medical Bioscience Laboratory, Yokohama City University Graduate School of Medical Life Science, 1-7-29 Suehiro-cho, Tsurumi-ku, Yokohama, 230-0045 Japan; 2 Graduate School of Agriculture and Life Sciences, The University of Tokyo, Tokyo, 113-8657 Japan

**Keywords:** Neuroscience, Psychology

## Abstract

The Nogo signal is involved in impairment of memory formation. We previously reported the lateral olfactory tract usher substance (LOTUS) as an endogenous antagonist of the Nogo receptor 1 that mediates the inhibition of axon growth and synapse formation. Moreover, we found that LOTUS plays an essential role in neural circuit formation and nerve regeneration. However, the effects of LOTUS on synapse formation and memory function have not been elucidated. Here, we clearly showed the involvement of LOTUS in synapse formation and memory function. The cultured hippocampal neurons derived from *lotus* gene knockout (LOTUS-KO) mice exhibited a decrease in synaptic density compared with those from wild-type mice. We also found decrease of dendritic spine formation in the adult hippocampus of LOTUS-KO mice. Finally, we demonstrated that LOTUS deficiency impairs memory formation in the social recognition test and the Morris water maze test, indicating that LOTUS is involved in functions of social and spatial learning and memory. These findings suggest that LOTUS affects synapse formation and memory function.

## Introduction

Overcoming the higher brain dysfunction that causes memory disorders, such as dementia, is an important issue currently being addressed worldwide. It is well known that cognitive decline occurs with aging in various higher brain functions, including spatial memory function^1^. The myelin-associated inhibitors (MAI), such as Nogo-A, myelin-associated glycoprotein (MAG), and oligodendrocyte myelin glycoprotein (OMgp), are potent inhibitors of axon regrowth^2–5^. The Nogo receptor-1 (NgR1) has been identified as a common receptor of these MAIs and adopts a co-receptor structure with the p75 neurotrophin receptor (p75NTR) and leucine-rich repeat and immunoglobin-like domain-containing Nogo receptor-interacting protein 1 (LINGO-1) or the tumor necrosis factor receptor superfamily (TROY), thus inducing a structural change in axons and spines via actin depolymerization through the activation of the Ras homolog family member A (RhoA)^6–8^. Nogo signaling inhibits axon regrowth through growth cone collapse, thereby inhibiting neural regeneration in the central nervous system via NgR1^9^. Conversely, Nogo signaling was also recently reported to reduce synaptic density in hippocampal primary cultured neurons^10,11^. Similarly, in the adult brain, NgR1-overexpressing mice exhibit a reduction of spine density and impairment of memory function^12,13^. In contrast, it was reported that the inhibition of Nogo signaling in hippocampal primary cultured neurons increased the number of synapses^14^. Furthermore, it has also clarified that spine density and memory function are increased in *nogo* gene KO mice^15^. Through such actions, Nogo signaling is considered to be a physiological factor that reduces memory function via the elimination of neural plasticity. Moreover, age-dependent increases in MAI and NgR1 expression has been reported in rats, which were accompanied by a decrease in memory function^16,17^. Recently, the leucine-rich glioma inactivated 1 (LGI1) and lateral olfactory tract (LOT) user substance (LOTUS) have been identified as antagonists of NgR1^18,19^. LGI1 was reported to be a secreted protein expressed in the CNS that contributes to synapse formation by inhibiting Nogo signaling^20–22^. LGI1 deficiency causes epileptic seizures, eventually leading to death by postnatal day 21. On the other hands, we previously discovered that LOTUS is a key factor involved in the formation of LOT, which is the secondary projection pathway for olfaction, and found that LOTUS functions as an endogenous antagonist of NgR1^19,23–25^. Although LOTUS is widely and abundantly expressed in the adult brain, its expression level in the hippocampus of rats decreases with age, and the memory function is reduced in correlation with the decrease in LOTUS expression^26,27^. However, the physiological role of LOTUS in synapse formation and memory function has not been clarified. Thus, the purpose of this study was to examine the involvement of LOTUS in synapse formation, spine morphology, and memory function.

## Results

### LOTUS is distributed in the synapse region of cultured hippocampal neurons

**Figure 1:**
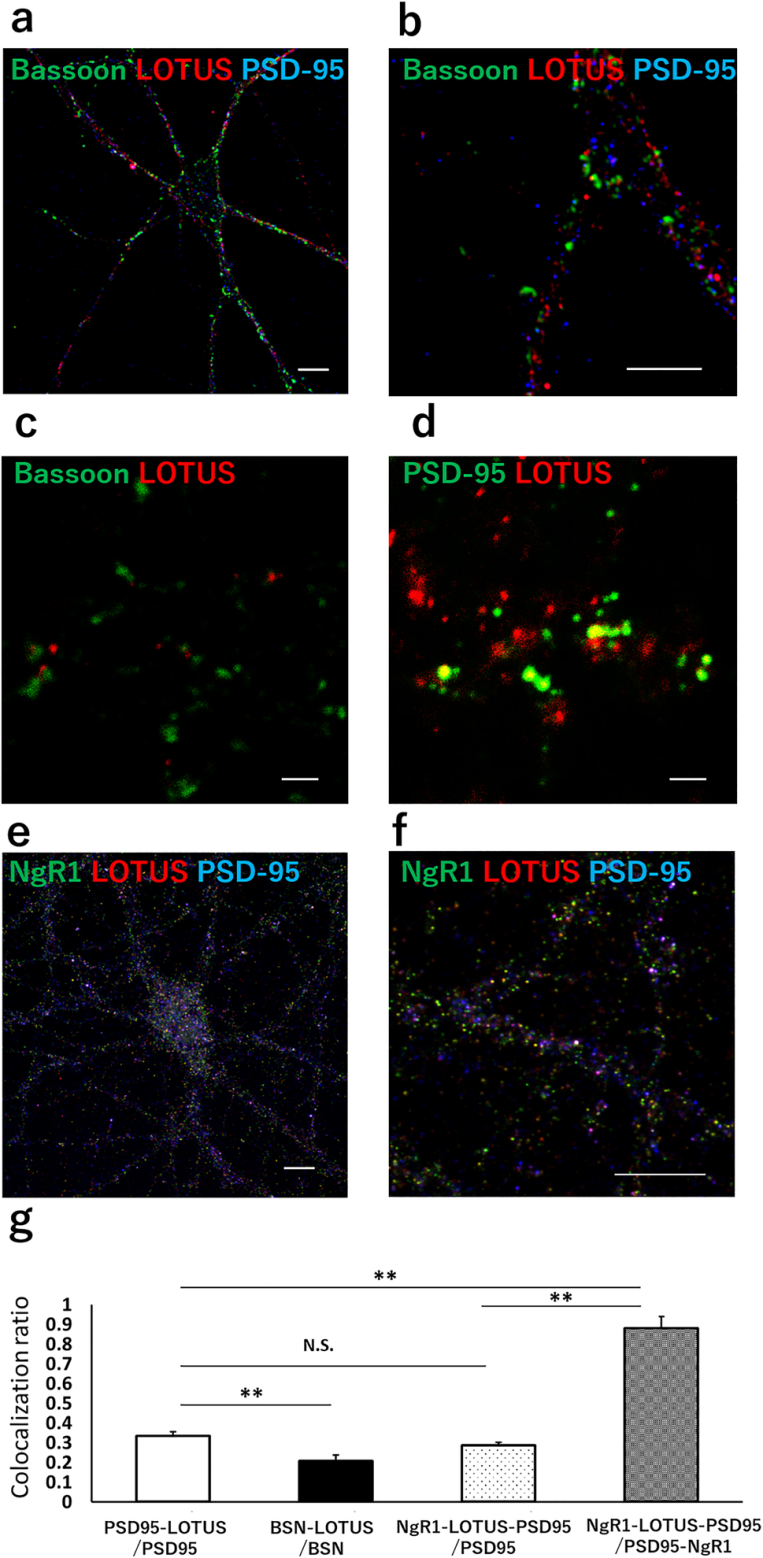
LOTUS is distributed in the synapse region. (**a**) LOTUS expression in cultured hippocampal neurons (DIV14). The image was acquired using a confocal microscope. PSD-95 (blue), LOTUS (red), and Bassoon (green). Scale bar, 10 µm. (**b**) Magnified image from (**a**). The segment was imaged at 5 × magnification. Scale bar, 5 µm. (**c**) Super-resolution image of Bassoon (green) and LOTUS (red) at the synapse site. The image was acquired using a STED microscope. Scale bar, 1 µm. (**d**) Super-resolution image of PSD-95 (green) and LOTUS (red) at the synapse site. The image was acquired using a STED microscope. Scale bar, 1 µm. (**e**) LOTUS and NgR1 localization in cultured hippocampal neurons (DIV14). The image was acquired using a confocal microscope. PSD-95 (blue), LOTUS (red), and NgR1 (green). Scale bar, 10 µm. (**f**) Magnified image from (**e**). The segment was imaged at 3 × magnification. Scale bar, 10 µm. (**g**) Quantification of LOTUS expression against that of Bassoon, PSD-95 and/or NgR1. Bars indicate ratio of the colocalization of PSD-95 and LOTUS in PSD-95 positive puncta (PSD95-LOTUS/PSD95), that of Bassoon and LOTUS in Bassoon positive puncta (BSN-LOTUS/BSN), that of NgR1, LOTUS and PSD-95 in PSD-95 positive puncta (NgR1-LOTUS-PSD95/PSD95) and that of NgR1, LOTUS and PSD-95 in PSD-95 and NgR1 positive puncta (NgR1-LOTUS-PSD95/PSD95-NgR1), respectively. Data are means ± SEM from 3–14 cells. **P < 0.01, one-way ANOVA post-hoc Tukey–Kramer.

NgR1 has been reported to be expressed at synapses and to negatively regulate synaptic morphology and density via the Nogo − NgR1 signal^10,12,14,15,28,29^. Here, first we examined the distribution of LOTUS in cultured hippocampal neurons using fluorescent immunostaining. We found that LOTUS was expressed in cellular regions that were co-stained with the Bassoon (a presynaptic marker) and postsynaptic density-95 (PSD-95, a postsynaptic marker), indicating that LOTUS is expressed in the synapse region of cultured hippocampal neurons (Fig. 1a − d). To assess the localization of LOTUS at the synapse, we compared LOTUS expression against that of Bassoon and PSD-95. We found that 33.5 ± 2.0% of PSD-95-positive puncta showed colocalization of PSD-95 and LOTUS, and 20.7 ± 3.1% of Bassoon-positive puncta showed colocalization of Bassoon and LOTUS (Fig. 1g). Next, we examined the localization of NgR1 and LOTUS at the post-synapse using fluorescent immunocytochemistry (Fig. 1e,f). We found that NgR1 was expressed in PSD-95-positive puncta, and that 28.6 ± 1.6% of PSD-95-positive puncta showed colocalization of LOTUS with NgR1. Moreover, 88.1 ± 6.1% of NgR1-positive, PSD-95-positive puncta showed colocalization of LOTUS with NgR1 (Fig. 1g). These findings suggest that LOTUS may be predominantly localized to the PSD-95-positive postsynapse and colocalizes with NgR1. Thus, LOTUS seems to be distributed in the synapse region of cultured hippocampal neurons.

### Loss of LOTUS decreases synaptic density in cultured hippocampal neurons

**Figure 2:**
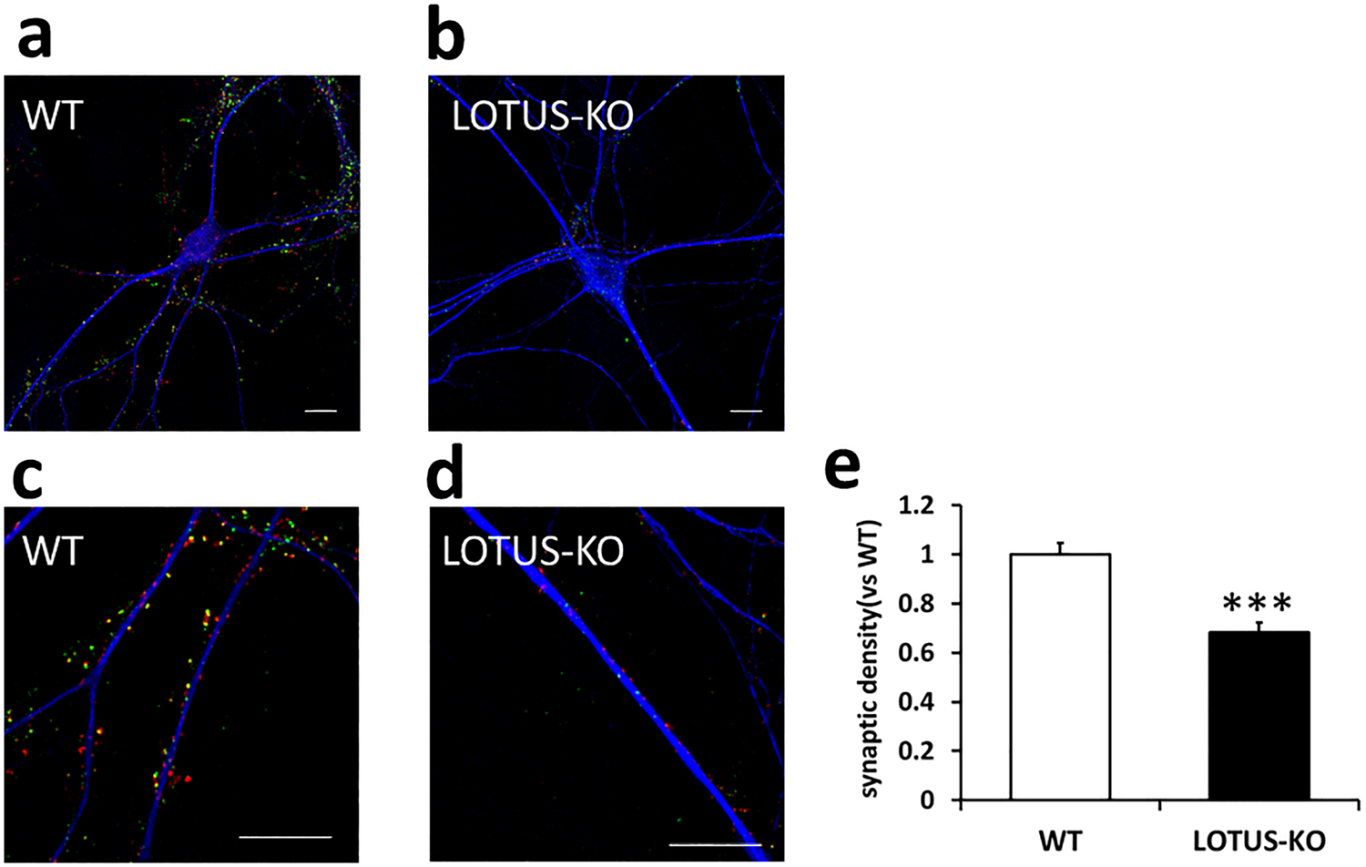
Loss of LOTUS decreases the density of PSD95/Bassoon puncta in cultured hippocampal neurons. (**a**, **b**) Cultured hippocampal neurons (DIV 14) derived from WT (**a**) and LOTUS-KO (**b**) mice. Neurons were immunostained with antibodies against Bassoon (red), PSD-95 (green), and MAP2 (blue). Scale bars, 10 µm. (**c**, **d**) Magnified images from (**a**) and (**b**). The segment was imaged at 3 × magnification. Scale bars, 10 µm. (**e**) Quantification of the synaptic density of Bassoon/PSD95 puncta along the dendrites of each neuron. Data were normalized to the synaptic density in WT neurons. Data are means ± SEM from four to five independent experiments. The total number of neurons analyzed (n) ranged from 16 to 20 cells per condition. ***P < 0.001, Student’s unpaired *t*-test.

To investigate the function of LOTUS in synapse formation, we measured the synaptic density via simultaneous immunostaining of the postsynaptic marker PSD-95 and the presynaptic marker Bassoon in cultured hippocampal neurons. The number of positive staining deposits was measured (Fig. 2a − d). The number of synapses was decreased in LOTUS-KO mice compared with WT mice (Fig. 2e). These data suggest that LOTUS contributes to synapse formation in cultured hippocampal neurons.

### Loss of LOTUS decreases dendritic spine density in the hippocampus of adult mice

**Figure 3:**
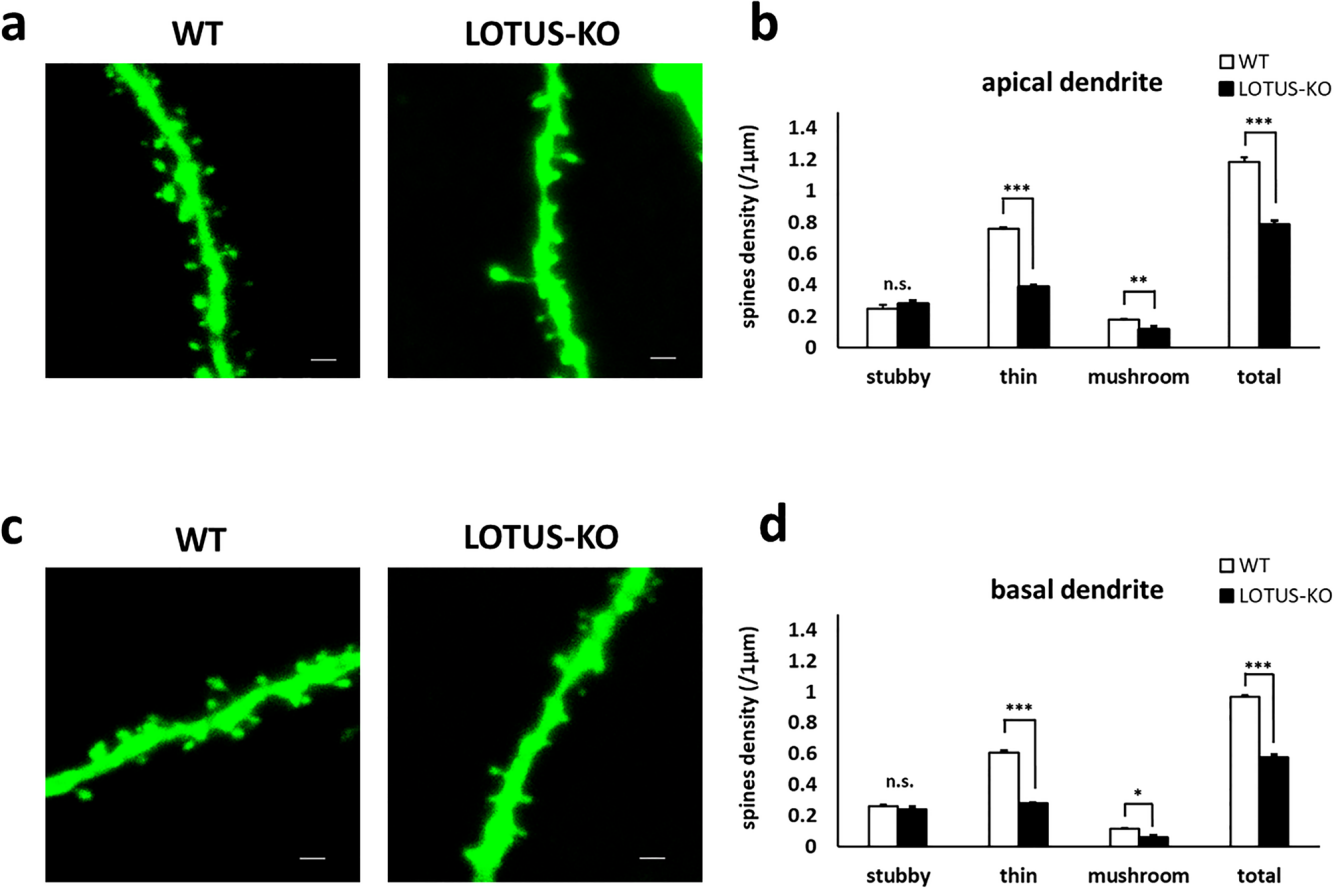
Loss of LOTUS decreases spine density in the hippocampal CA1 region. (**a**) LOTUS controls the dendritic spine in apical dendrites. Scale bars, 1 µm. (**b**) Quantification of the spine density in apical dendrites of hippocampal neurons in the CA1 region; 41 − 43 dendrites were analyzed in each mouse (WT: n = 3; LOTUS-KO: n = 3). Data are means ± SEM from **P < 0.01, ***P < 0.001. Student’s unpaired *t*-test. (**c**) LOTUS controls the dendritic spine in basal dendrites. Scale bars, 1 µm. (**d**) Quantification of the spine density in basal dendrites of hippocampal neurons in the CA1 region; 40 − 44 dendrites were analyzed in each mouse (WT: n = 3; LOTUS-KO: n = 3). Data are means ± SEM from *P < 0.05, ***P < 0.001, Student’s unpaired *t*-test.

Because LOTUS-KO mice showed a decrease in synaptic density in cultured hippocampal neurons (Fig. 2), next we investigated role of LOTUS in dendritic spine morphology in the adult hippocampus. Mice in which dendritic spines can be visualized were created using Thy1-EGFP mice. Thus, the spine density of EGFP-positive dendrites was examined in the hippocampus of adult Thy1-EGFP WT and Thy1-EGFP LOTUS-KO mice. The apical dendritic spine density in hippocampal CA1 pyramidal neurons was significantly decreased in LOTUS-KO mice compared with WT mice. In particular, the number of mushroom-type and thin-type spines was significantly decreased in LOTUS-KO mice. However, no difference was observed in the number of stubby-type spines in these mice (Fig. 3a,b). Similarly, the same measurement was performed in basal dendrite, which yielded similar results to those obtained for apical dendrites (Fig. 3c,d). These results indicate that loss of LOTUS reduces the number of thin and mushroom-type spines in hippocampal CA1 pyramidal neurons, as well as the total spine density, suggesting that LOTUS may contribute to hippocampal synapse formation.

### Loss of LOTUS impairs hippocampus-dependent memory formation

**Figure 4:**
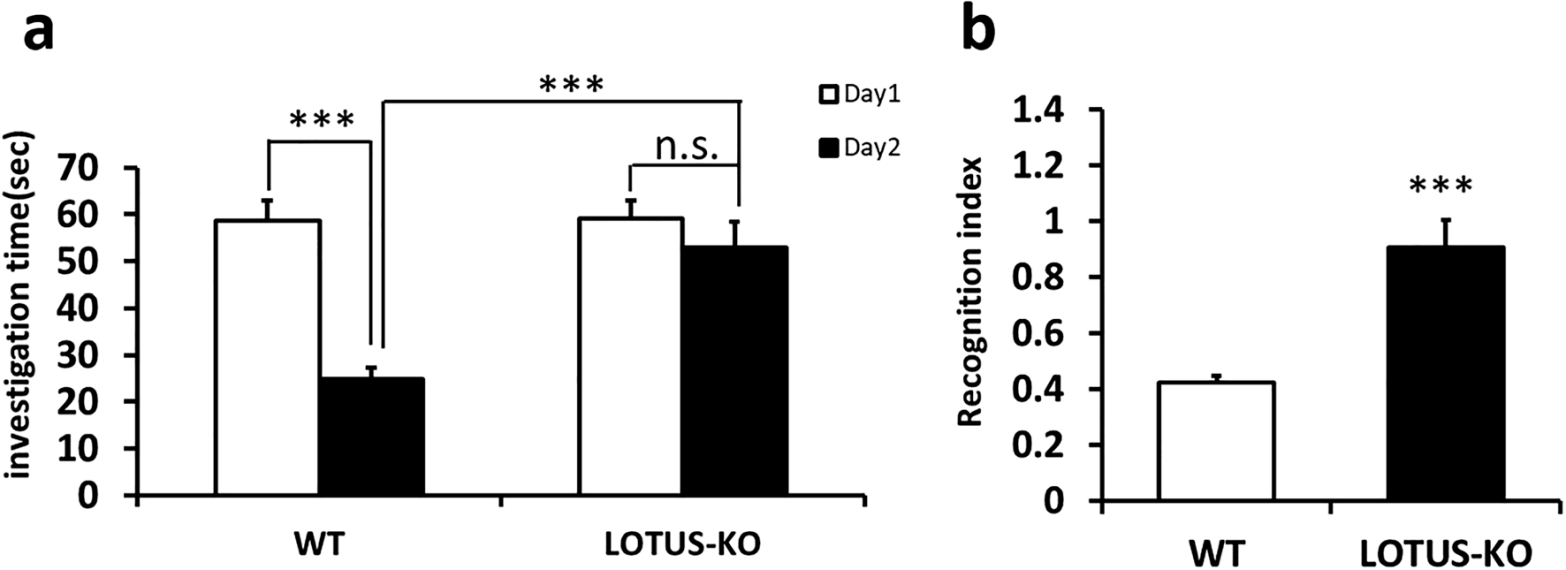
Loss of LOTUS causes impairment in social cognitive memory. (**a**) Comparison of the social investigation time during 3 min of exposure time. Data are means ± SEM from WT mice (n = 9) and LOTUS-KO mice (n = 10). ***P < 0.001, Day1 versus Day2, Student’s paired *t*-test, WT mice versus LOTUS-KO mice, Student’s unpaired *t*-test. (**b**) Recognition index. Data are means ± SEM from WT (n = 9) and LOTUS-KO (n = 10) mice. ***P < 0.001, Student’s unpaired *t*-test.

**Figure 5:**
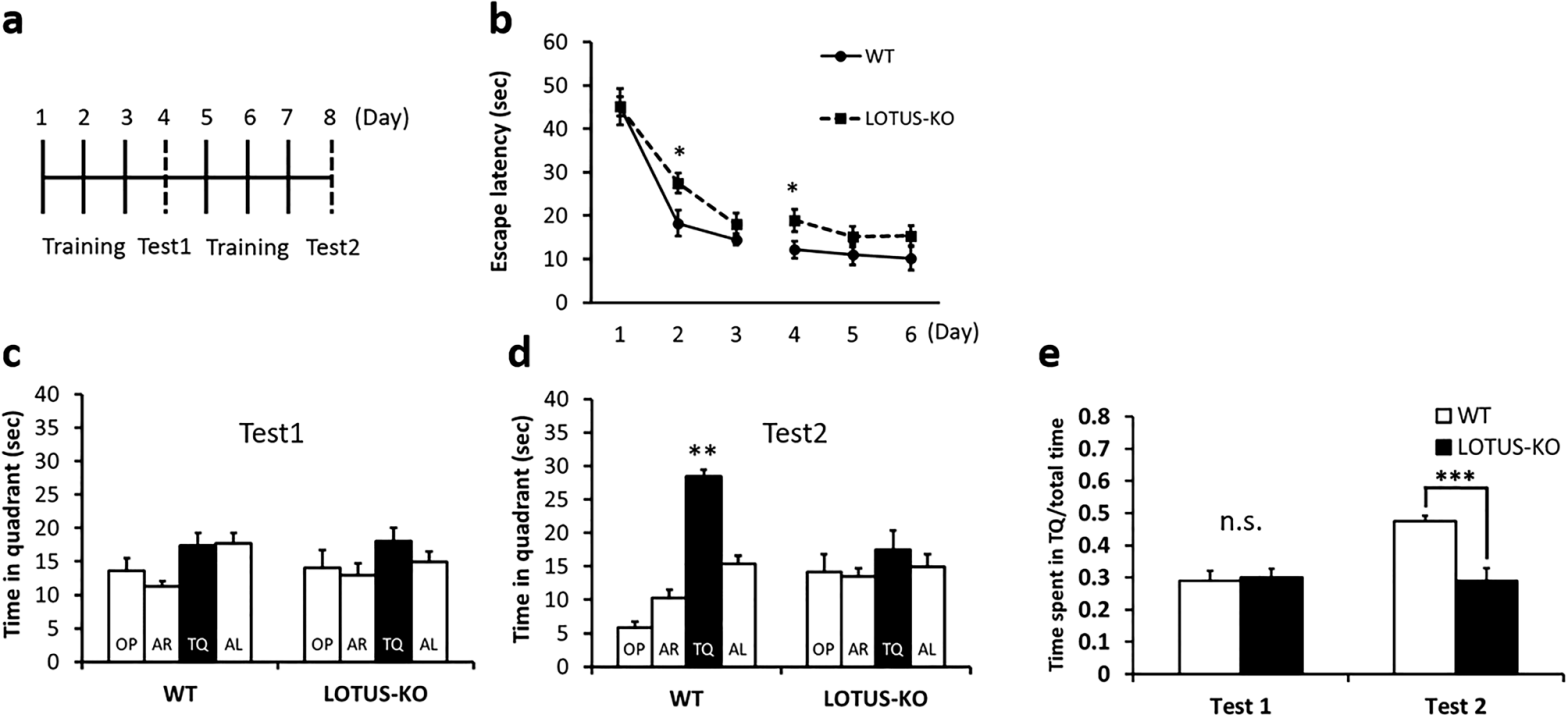
Loss of LOTUS causes impairment in spatial learning. (**a**) Time course of the experimental procedure of the Morris Water Maze test. (**b**) Escape latency time in WT and LOTUS-KO mice. Data are means ± SEM from WT mice (n = 15) and LOTUS-KO mice (n = 13). *P < 0.05, Student’s unpaired *t*-test. (**c**) Test in WT and LOTUS-KO mice after training for 3 days. Data are analyzed by χ^2^ test. (**d**) Test in WT and LOTUS-KO mice after training for 6 days. Data are means ± SEM from WT (n = 15) and LOTUS-KO (n = 13) mice. **P < 0.01, one-way ANOVA with post-hoc Steel − Dwass test and with χ^2^ test. Opposite (OP); adjacent right (AR); target quadrant (TQ); adjacent left (AL). (e) Ratio of time spent in the TQ Ward during the test. Data are means ± SEM from WT (n = 15) and LOTUS-KO (n = 13) mice. ***P < 0.001, Student’s unpaired *t*-test.

To investigate whether LOTUS deficiency affects learning and memory, we first evaluated the ability of social cognitive memory formation in WT and LOTUS-KO mice. The social recognition test is a behavioral analysis that evaluates hippocampal-dependent social cognitive memory formation in mice. In this experiment, a mature test mouse encounters a juvenile mouse as a stranger for 3 min; 24 h later, it is determined whether the test mouse remembers the juvenile mouse. A significant reduction in the investigation time was observed in WT mice (n = 9), while no significant decrease was observed in LOTUS-KO mice (n = 10) (Fig. 4a). Furthermore, the results of the recognition index, which indicates the ratio of the social investigation times during the Day2 and Day1, meant that LOTUS-KO mice showed a significantly worse recognition compared with WT mice (Fig. 4b), suggesting that LOTUS-KO mice have impaired memory compared with WT mice. Thus, these data show that loss of LOTUS impairs social-recognition-related memory. Next, to evaluate the ability of spatial learning and memory, we performed the Morris water maze test, which is a behavioral analysis that evaluates hippocampal-dependent spatial memory formation in mice (Fig. 5a). In 6 days training, the escape latency was significantly longer in LOTUS-KO mice (n = 15) compared with WT mice (n = 13; two-way repeated-measures ANOVA). In addition, LOTUS-KO mice had significantly longer escape latency at Day 2 and Day 4 compared to WT mice (Student’s unpaired *t*-test, Fig. 5b). In test 1, after training twice a day for 3 days, the WT and LOTUS-KO mice did not show any difference in the staying time in the target quadrant (TQ) (by χ^2^ test, Fig. 5c), where the platform was set. In test 2, after training for 6 days, the staying time in the TQ was significantly increased in WT mice, whereas no difference was observed in LOTUS-KO mice (by χ^2^ test, Fig. 5d). Furthermore, the ratio of time spent in the TQ during the probe test was not significantly different between WT and LOTUS-KO mice in Test 1, whereas a significant reduction in this ratio was observed in LOTUS-KO mice in Test 2 compared with WT mice (Fig. 5e). No difference was observed in the body weight and swimming speed of LOTUS-KO mice compared with WT mice (Fig. S2a,b). These results suggest that LOTUS-KO mice show an impairment of spatial learning and memory. Taken together, the results imply that loss of LOTUS impairs the ability to form hippocampus-dependent memory, such as social cognitive and spatial memories, and that LOTUS may be required for memory formation.

## Discussion

Nogo signaling inhibits neurite outgrowth with growth cone collapse, thereby inhibiting axonal regeneration after injury in the central nervous system via NgR1^2,3,6,9^. MAIs such as Nogo-A, MAG, and OMgp are potent inhibitors of axon regrowth as ligands of NgR1^4,5^. NgR1 adopts a co-receptor structure with p75NTR and LINGO-1 or TROY and induces structural changes in the cytoskeleton through activation of RhoA^7,8^. In this context, it was reported that Nogo signaling reduces synaptic density in the hippocampus^14,15^. Furthermore, it was also reported that the *β*-amyloid protein (A*β*), which is believed to be a causal protein of Alzheimer’s disease, also binds to NgR1 and reduces synaptic density, thereby inducing defective memory function^30^. In each case, NgR1 leads to the inhibition of synapse formation via the RhoA − ROCK signal. As an NgR1 antagonist, LGI1 has been reported to contribute to synapse formation by inhibiting Nogo signaling^18,20–22^. Therefore, we hypothesized that LOTUS, an endogenous antagonist of NgR1, is also involved in synapse formation and memory formation. To address this issue, we first examined the location of LOTUS expression in cultured hippocampal neurons using fluorescent immunocytochemistry. The data showed that LOTUS was distributed along dendrites and in synapse regions of hippocampal neurons. It has been reported that inhibition of NgR1 by shRNA increased synaptic density in cultured hippocampal neurons^14^. Moreover, the application of Nogo to cultured hippocampal neurons has been reported to reduce synaptic density^10,11^. In the present study we observed LOTUS co-localizing with NgR1 at PSD-95-positive synaptic sites, indicating that the interaction between NgR1 and LOTUS in the synapses of hippocampal neurons may suppress Nogo signaling and affect synapse formation. This idea is supported by data showing that the loss of LOTUS decreases synapse density in cultured hippocampal neurons and decreases dendritic spine density in the adult hippocampus*.*

Interestingly, LOTUS-KO mice exhibited a decreased density of thin- and mushroom-type spines compared with WT mice. These data suggest that LOTUS may influence synaptic morphology. As Nogo signaling is known to regulate actin dynamics^7^, it is possible that LOTUS plays a role in maintaining the synaptic actin assembly, supporting synaptic maturation and morphology.

It has also been reported that suppression of Nogo signaling increases hippocampal-dependent long-term memory function, and that enhancement of Nogo signaling decreases memory formation^12,15^. Based on these findings, we evaluated hippocampal-dependent long-term memory formation in LOTUS-KO mice; we found that social cognitive memory and spatial learning and memory were impaired in the absence of LOTUS in these animals, suggesting that LOTUS may contribute to hippocampus-dependent memory formation. Future studies of activity-dependent dendritic spine dynamics in LOTUS-KO mice are required to fully understand the contribution of LOTUS to synaptic plasticity.

It has been reported that the increase in Nogo signal^16,17,27^ or the decrease of LOTUS expression^26^ in the hippocampus according to aging causes memory impairment. Both processes may have a synergistic effect on age-dependent memory impairment. It would be interesting to examine whether LOTUS overexpression or the blockade of the decrease in LOTUS expression suppress the age-dependent memory impairment. Conversely, NgR1 has been reported as a receptor of the A*β* protein, a causative protein of Alzheimer’s disease; moreover, A*β* binding to NgR1 suppresses synapse formation, and A*β* action through NgR1 may affect synaptic plasticity and cause memory impairment eventually^30,31^. Recently, we found that LOTUS also binds to the paired immunoglobulin-like receptor B (PirB) and suppresses Nogo-induced PirB function^32^. PirB also acts as an A*β* receptor, and A*β* binding to PirB impairs memory function^33^. Therefore, whether LOTUS is involved in a regulatory function in the binding of A*β* to NgR1 or PirB is a fascinating subject for future research. Further investigations are required to identify the effective functions of LOTUS in the development of preventive and therapeutic approaches for senile amnesia and Alzheimer’s disease.

## Methods

### Animals

C57BL/6 J mice were purchased from Charles River Co. (Japan, Inc.), and the *lotus/crtac1b* gene knockout mice (Acc. No. CDB0599K,(http://www.cdb.riken.jp/arg/mutant%20mice%20list.html) were generated as previously described^19^ (http://www.cdb.riken.jp/arg/Methods.html). The heterozygous Thy1-EGFP mice were maintained by crossing with wild-type (WT) C57BL/6 J mice^34^. The mice were housed in a standard mouse facility and were provided autoclaved diet and water. Throughout the experimental procedures, all efforts were made to minimize the number of animals used and their suffering. The experimental procedures were approved by the institutional animal care and use ethical committee of Yokohama City University and were carried out in accordance with the recommended guidelines. The lotus/crtac1b mutants were assessed on the C57BL/6 J background.

### Cell culture of hippocampal neurons

The hippocampal nerve cell primary culture method was partially modified from the original protocol^35^. The hippocampus was excised from embryos (E17.5) of WT and LOTUS-KO mice. Pregnant mice of each genotype were deeply anesthetized with isoflurane (Pfizer) and the embryos were removed. The hippocampus was dissected and dispersed using 0.25% trypsin and 100 µg/ml DNase at 37 °C for 12 min. Dispersed cells were immersed in a 24-well dish (Greiner Bio-One). The glass cover slips (*φ*, 12 mm; Matsunami) were coated with 10 μg/ml of polyethyleneimine and 10 μg/ml of laminin, and the surface was seeded with 0.5 × 10^5^ cells/dish. Neurobasal medium (Gibco) containing 10% fetal bovine serum (Biowest) was used as the plating medium, and 1 × B-27 (Gibco), 1 × Glutamax (Gibco), and Neurobasal medium (Gibco) were used as the culture medium.

### Immunohistochemistry

After washing with phosphate-buffered saline (PBS), the hippocampal primary cultured neurons (DIV 14 days) were fixed with methanol at − 20 °C for 8 min, then washed with PBS and treated with 1% bovine serum albumin (BSA) (Nacalai Tesque) in PBS for 20 min. The primary antibodies (monoclonal hamster antibodies against LOTUS (H24G11-MAB) at a dilution of 5 µg/ml (Fig. 1c,d) or 1 µg/ml (Fig. 1a,b,e,f) and goat anti-NgR1 (1/500; R&D) were applied before fixation, as described previously^19^, followed by cell fixation with 4% paraformaldehyde at 37 °C for 10 min and at RT for 10 min. Mouse anti-PSD-95 (1/1000; Invitrogen), rabbit anti-Bassoon (1/1000; Synaptic systems), and chicken anti-MAP2 (1/1000; Abcam) antibodies diluted with 1% BSA/PBS were used as primary antibodies. Cells were incubated with the primary antibody for 1 h. After washing with PBS, Alexa Fluor 488-conjugated goat anti-mouse IgG (1/2000; Invitrogen) diluted with 1% BSA/PBS, Alexa Fluor 488-conjugated donkey anti-goat IgG (1/1000; Invitrogen), Alexa Fluor 532-conjugated goat anti-rabbit IgG (1/100; Invitrogen) (Fig. 1c), Alexa Fluor 532-conjugated goat anti-mouse IgG (1/100; Invitrogen) (Fig. 1d), Alexa Fluor 568-conjugated goat anti-hamster IgG (1/100; Invitrogen) (Fig. 1c − d), Alexa Fluor 594-conjugated goat anti-rabbit IgG (1/2000; Invitrogen), Alexa Fluor 594-conjugated sheep anti-hamster IgG (1/1000), Alexa Fluor 647-conjugated donkey anti-mouse IgG (1/2000) and Alexa Fluor 647-conjugated goat anti-chicken IgY (1/2000; Invitrogen) were used as the secondary antibodies and incubated with the cells at room temperature for 1 h. Subsequently, the cells were washed with PBS and mounted with cover slips using Fluoromount (CosmoBio). Fluorescence images were obtained using a TCS SP8 microscope (Leica) equipped with a 100 × oil-immersion objective lens (NA, 1.4) and LAS X software (Leica). Super-resolution images were acquired using STED mode; confocal images were acquired using normal confocal mode. All images were captured at a resolution of 1024 × 1024 pixels with a z-step of 0.5 µm (Fig. 1a–f). The analysis for synaptic marker quantification was performed with Leica LAS X and analyzed on flattened Z-projections. All captured images were subjected to a luminance histogram threshold with LAS X (luminance for PSD-95: 30–150; Bassoon: 30–150; LOTUS: 50–120; NgR1: 30–120). The positive clusters with PSD-95 and LOTUS colocalization, Bassoon and LOTUS colocalization, NgR1, LOTUS and PSD-95 colocalization, and PSD-95 and NgR1 colocalization within 40 µm from the branch point close to the cell body were counted, respectively.

### Analysis of synapse density in cultured hippocampal neurons

All fluorescence immunostaining images were acquired using a confocal microscope (TCS SP8; Leica) and the LasX software (Leica). The segment was imaged at 1–3 × magnification. All images were taken by using a resolution of 1024 × 1024 pixels with a z-step of 0.5 µm. Independently observable immunostaining with anti-PSD-95, anti-Bassoon, and anti-MAP2 antibodies was examined to identify the synapse sites (Fig. 2a,b). All captured images were subjected to a luminance histogram threshold with LAS X (luminance for PSD-95: 30–150; Bassoon: 30–150; MAP2: 10–200). The positive clusters with PSD-95 and Bassoon colocalization within 40 µm from the branch point close to the cell body of each MAP2-positive dendrite were counted, and the positive deposits within 10 µm of the proximal dendrite were measured.

### Analysis of dendritic spine density in the adult hippocampus

Mice (male, 2 months old) were deeply anesthetized with isoflurane (Pfizer) and perfused with 4% paraformaldehyde. The brain was then removed and fixed overnight in the same fixative. Subsequently, the fixed brain was immersed in 30% sucrose and stored at − 80 °C. Coronal Sects. (30 µm) were prepared using a cryostat. Fluorescence images were acquired using a confocal microscope (TCS SP8; Leica) equipped with a 63 × (NA, 1.4) oil-immersion objective and the LAS X software (Leica). Images were captured at a resolution of 512 × 512 pixels with a z-step of 0.5 µm. The confocal stack was semi-automatically analyzed with the Neuron Studio software^36^ (http://research.mssm.edu/cnic/). Spine density was calculated as the number of spines divided by the length of the dendrite segment. Stubby spines were identified by a head-to-neck diameter ratio less than 1.1. Thin spines were determined by a head-to-neck diameter ratio greater than 1.1 and a maximum head diameter less than 0.35 µm. Mushroom spines were determined by a head-to-neck diameter ratio greater than 1.1 and a maximum head diameter greater than 0.35 µm. Spine density was quantified on the first branching site of apical or basal dendrites from hippocampal CA1 pyramidal neurons.

### Behavioral tests

Before performing behavioral analysis, 3 min handling was performed for 5 days. The social recognition test and the Morris water maze test were performed using different mice, as described below.

#### Social recognition test

The social recognition test is a behavioral analysis that measures social cognitive memory, which is a hippocampal-dependent type of memory^37,38^. First, as a training session, juvenile mice (male, 2 − 3 weeks old) were used as strangers who had never met the test mature test mouse (male, 2 months old); the test mouse encountered the juvenile mouse for 3 min, during which the contact between the two mice via sniffing was measured as the time required for individual recognition. Twenty-four hours later, the investigation time was measured again in the same combination of mature and juvenile mice, as a test. When the investigation time at the time of testing was significantly decreased compared with that at the time of training, we considered that the mature test mouse remembered and recognized the juvenile mouse.

#### Morris water maze test

The Morris water maze test is used to examine whether a test mouse undergoes spatial learning^39^. The mice (male, 2 months old) were trained with two trials per day at an interval of 1 min for 6 days. The mice were trained at approximately the same time every day. In the probe test, at 24 h after training on days 3 and 6, the platform was removed, and the mice were allowed to swim for 1 min. The time spent in each quadrant (opposite [OP], adjacent right [AR], target quadrant [TQ], and adjacent left [AL]) was measured and compared.

### Statistical analysis

The J-STAT software was used for statistical analysis. All data are expressed as the mean ± standard error. The colocalization ratio was analyzed by one-way ANOVA post hoc Tukey–Kramer (Fig. 1g). The escape latency of the Morris water maze was analyzed by two-way repeated ANOVA (Fig. 5b). The staying time in the TQ of the Morris water maze was analyzed by χ^2^ test and one-way ANOVA with post-hoc Steel − Dwass test (Fig. 5c,d). Differences were considered significant at *P* < 0.05.

## Supplementary information

Supplementary Figure S1.

Supplementary Figure S2.

Supplementary information.
