# Supplementary figures and images for: LOTUS, an endogenous Nogo receptor antagonist, is involved in synapse and memory formation
Source: Sci Rep. 2021 Mar 3;11:5085. doi: 10.1038/s41598-021-84106-y (PMC7930056; doi:10.1038/s41598-021-84106-y)

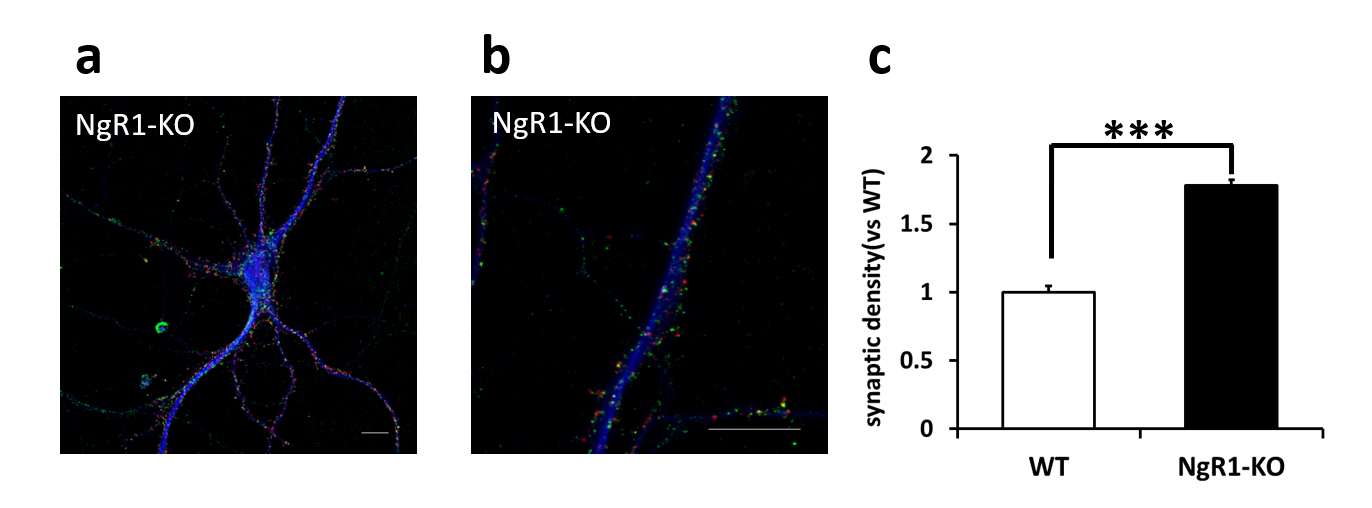

Supplement: Supplementary file 1 — Supplementary Figure S1. [file 41598_2021_84106_MOESM1_ESM.tif]

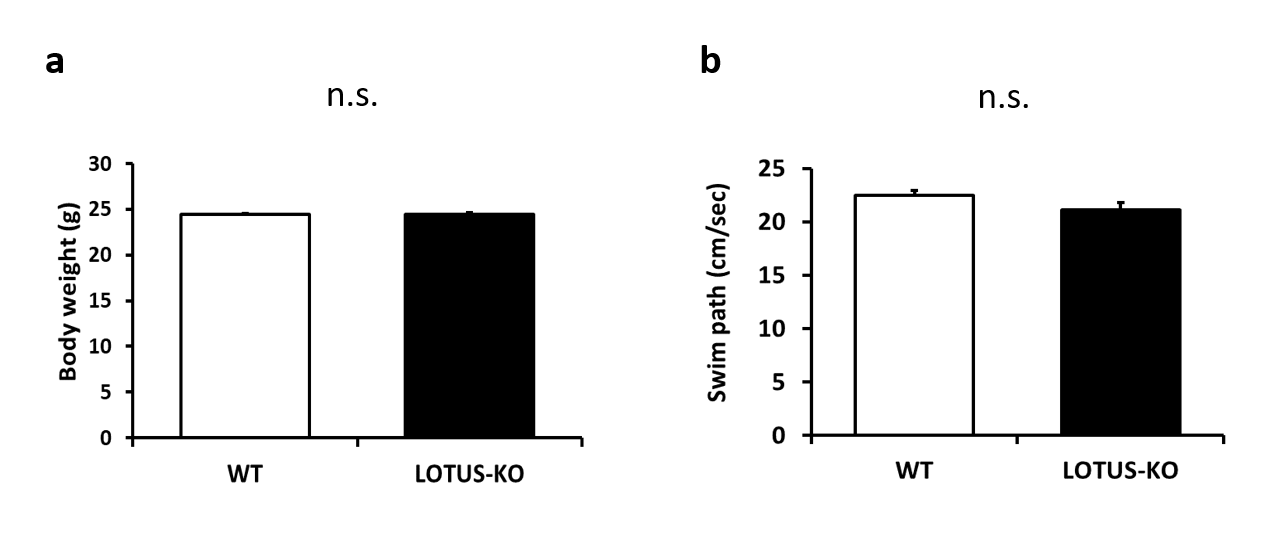

Supplement: Supplementary file 2 — Supplementary Figure S2. [file 41598_2021_84106_MOESM2_ESM.tif]
