# Supplementary material for: LOTUS, an endogenous Nogo receptor antagonist, is involved in synapse and memory formation
Source: Sci Rep. 2021 Mar 3;11:5085. doi: 10.1038/s41598-021-84106-y (PMC7930056; doi:10.1038/s41598-021-84106-y)
Supplement: Supplementary file 3 — Supplementary information. [file 41598_2021_84106_MOESM3_ESM.docx]

**Supplementary information**

Title:

LOTUS, an endogenous Nogo receptor antagonist, is involved in synapse and memory formation

# Authors:

# Ryohei Nishida^1^, Yuki Kawaguchi^1^, Junpei Matsubayashi^1^, Rie Ishikawa^2^, Satoshi Kida^2^, and Kohtaro Takei^1,*^

^1^Molecular Medical Bioscience Laboratory, Yokohama City University Graduate School of Medical Life Science, Yokohama, 230-0045, Japan.

^2^Graduate School of Agriculture and Life Sciences, The University of Tokyo, Tokyo, 113-8657, Japan.

Corresponding author:

*Kohtaro Takei, Molecular Medical Bioscience Laboratory, Yokohama City University Graduate School of Medical Life Science, 1-7-29 Suehiro-cho, Tsurumi-ku, Yokohama 230-0045, Japan, TEL: +81-45-508-7240, E-mail: [kohtaro@yokohama-cu.ac.jp](mailto:Kohtaro@yokohama-cu.ac.jp).

**Supplementary Figure S1**

Loss of NgR1 increases synaptic density in cultured hippocampal neurons.

(a) Cultured hippocampal neurons (DIV 14) derived from NgR1-KO mice. Neurons were immunostained with antibodies recognizing Bassoon (red), PSD-95 (green), and MAP2 (blue). Scale bar, 10 µm.

(b) Magnified images from (b). Scale bar, 10 µm.

(c) Quantification of the synaptic density of Bassoon/PSD95 puncta along the dendrites of each neuron. Data are normalized to the synaptic density in WT neurons. Data are means ± SEM from three to four independent experiments. The total number of neurons analyzed (n) ranged from 12 to 16 cells per condition. ****P* < 0.001, Student’s unpaired *t*-test.

**Supplementary Figure S2**

(a) Comparison of body weight. There was no significant difference between WT and LOTUS-KO mice. Data are means ± SEM from WT (n=43) and LOTUS-KO (n=39) mice. Student’s unpaired *t*-test.

(b) Comparison of swimming ability. There was no significant difference between WT and LOTUS-KO mice. Data are means ± SEM from WT (n=15) and LOTUS-KO (n=13) mice. Student’s unpaired *t*-test.
